# Emotion-Bracelet: A Web Service for Expressing Emotions through an Electronic Interface

**DOI:** 10.3390/s16121980

**Published:** 2016-11-24

**Authors:** Alicia Martinez, Hugo Estrada, Alejandra Molina, Manuel Mejia, Joaquin Perez

**Affiliations:** 1 Computer Science Department, National Centre for Research and Technology Development, Cuernavaca 62490, Mexico; amolina@cenidet.edu.mx (A.M.); mlavalle@cenidet.edu.mx (M.M.); jperez@cenidet.edu.mx (J.P.); 2 INFOTEC Center for Research and Innovation in Information Technology and Communications, Mexico City 14050, Mexico; hugo.estrada@infotec.mx

**Keywords:** the Emotion-Bracelet, polarity, emotions

## Abstract

The mechanisms to communicate emotions have dramatically changed in the last 10 years with social networks, where users massively communicate their emotional states by using the Internet. However, people with socialization problems have difficulty expressing their emotions verbally or interpreting the environment and providing an appropriate emotional response. In this paper, a novel solution called the Emotion-Bracelet is presented that combines a hardware device and a software system. The proposed approach identifies the polarity and emotional intensity of texts published on a social network site by performing real-time processing using a web service. It also shows emotions with a LED matrix using five emoticons that represent positive, very positive, negative, very negative, and neutral states. The Emotion-Bracelet is designed to help people express their emotions in a non-intrusive way, thereby expanding the social aspect of human emotions.

## 1. Introduction

Emotion is a subjective experience that is accompanied by biological and behavioral changes as well as, physiological responses. In several theories, physiological arousal, expressive behaviors, and conscious experiences are distinguished as part of human emotion. 

People with socialization problems have difficulty expressing their emotions verbally or interpreting the environment and providing an appropriate emotional response [1]. Currently, several research works have focused on improving interpersonal communication through the development of devices using various technologies and techniques such as data from motion sensors [1,2,3] and brain waves [4]. Moreover, with the use of new technologies such as Social Network Sites (SNS), intepersonal communication has increased. Social networking websites such as Facebook are popular platforms where individuals can easily share opinions, thoughts, emotions, and personal activities with family, friends, and acquaintances. Social media networks have completely changed the way in which people have relationships and communicate emotions. In the past, the communication of an emotion was made face-to-face, currently, people use social networks to massively communicate their emotions. Currently, there are studies that support the analysis of communication media for dealing with people’s emotions [5]. There are companies paying attention to the analysis of sentiment related to their brands and products in social media, as well as in designing advertising content that triggers emotions [6]. Other marketing companies analyze how social media interactions with tourism brands affect how consumers think and feel about their brands [7]. Finally, there are research works that predict the user’s personality through the publicly available information on their Facebook profile [8].

In spite of the great amount of information that is shared with people on SNS and the ease of access to these sites, the creation of mechanisms for the analysis of this information is a top priority, since on many occasions, emotions can be hidden in the text with no explicit reference to words that directly denote them—such as fear, happiness, or sadness. Therefore, mechanisms are needed to detect the emotions that are expressed in text messages of SNS in order to make explicit them.

Therefore, our proposed solution is composed of two contributions: (a) a Web Service called SWePT that permits the detection of the polarity of texts (in Spanish) from SNS; and (b) the design of a graphic interface (the Emotion-Bracelet), which shows the emotional state presented in the texts published by a single user in real time.

The rest of the paper is structured as follows: Section 2 presents the background and related work. Section 3 presents the SWePT method to obtain the polarity of a SNS comment. Section 4 presents the SWePT service implementation. Section 5 presents the design, the construction of the Emotion-Bracelet, and the case study. Section 6 presents the evaluation and results. Finally, Section 7 presents the conclusions and lines of future work.

## 2. Background and Related Works

The main concepts used in this paper are presented in this section: emotion, sentiment analysis, and opinion polarity. This section also presents the related works.

### 2.1. Emotion

An emotion is a complex state that is characterized by excitement or disturbance that predisposes to action. Emotions are generated in response to an external or internal event [9].

Depending on how the antecedent (event) can affect a person, emotions can be positive or negative, this does not mean they are good or bad, but rather that emotions are positive or negative depending on the comfort or discomfort they cause in the person [10].

Negative emotions are the result of an unfavorable evaluation regarding our well-being. There are various forms of threat, frustration, loss, etc.—these include fear, anger, sadness, guilt, shame, envy, jealousy, disgust, etc. Positive emotions are the result of a favorable evaluation regarding the achievement of our well-being. They include joy, love, affection, relief, etc.

### 2.2. Sentiment Analysis

Opinion mining (also known as sentiment classification or subjectivity analysis) refers to a broad area of natural language processing and text mining. It is not concerned with the topic that a document is about, but rather with the opinion it expresses. In other words, its aim is to determine the attitude (feelings, emotions, and subjectivities) of a speaker or a writer with respect to a topic. One of the major tasks in opinion mining is the classification of the opinion’s polarity, which consists of determining whether the opinion is positive, negative, or neutral [11].

### 2.3. Opinion Polarity

The semantic orientation of a word or set of words, that indicates that if the value is positive it is considered as a favorable opinion. If the value is negative, it indicates an unfavorable opinion. Different absolute values of the measure also report *n* different degrees of intensity in these implications [12].

The polarity is also defined as a value component of language (i.e., those linguistic aspects that encode subjectivity in terms of the positivity or negativity of the speaker with respect to the topic) [13].

### 2.4. Related Works

Different studies focus on the development of devices for improving interpersonal communication. For example, a study is presented in [14], that examined the use of a mobile phone application that combines experience sampling of mood with exercises inspired by cognitive behavioral therapy. The authors designed a touch screen Mood Map and several single-dimensional mood scales. This interface is based on the circumflex model, which describes all emotions by these two factors. Another study is presented in [15], which examines the effects of interface proximity. This proximity is conceptualized as near, moderate, and far based on participants’ anxiety when they receive news such as crime alerts. Interface proximity uses three different media platforms: stationary (desktop computer), portable (laptop computer), and ubiquitous (hand-held devices). 

Lumitouch [3] uses interactive picture frames with colored LEDs for the exchange and sharing of emotions in real time, between two people who are geographically distant. When a person wants to share his feelings, pressure the screen and depending on the pressure, the portrait of the other person is illuminated with a specific color. Similarly, Nimio [16] is a device that represented in different ways (a pyramid, a cube, a triangular prism, a square prism) and with three colors (red, green and blue). The purpose of this device is to support the exchange of activities among a group of collaborators, who are physically distributed throughout the company; when a user wants to communicate an activity to his/her team they move the device to activate it. Then he or she activates a color on the device and all the devices with the same color will begin to flash.

Several devices have been developed that are able to express emotions from physical movements made by the user. An example of this research area is the Emotion Caster [2], which uses a penguin puppet that shows a facial expression indicating emotion. The emotions that can be represented are: happiness, frustration, anger, and sadness. The Emoti-Ring [17] is a ring-shaped device that allows users to express their emotions and perceive the emotions of a predefined contact. The user rotates the ring to select the desired emotion, and it automatically transmits the emotion to a mobile device via Bluetooth. The software application then transmits it to a SNS where the emotion is published. Emoti-Ring also gets the emotional state of a specific friend through a SNS service and displays the emotion on a screen. Picture Emoti [1] is a picture frames that enables the user to transmit his/her emotional state by pressing emotion buttons, that use a color-emotion code (white for neutral, yellow for happiness, blue for melancholy, grey for sad, red for angry).

Other devices have been designed to improve communication for couples. Such is the case of Lover’s Cup [18], which are two cups paired via wireless that are able to emulate the interaction that exists in a couple while enjoying a drink. When a person holds one of the cups, the cup of the partner glows dimly. When both partners of the take the cups at the same time, both cups are highly illuminated. Calmate [19] improves the communication between couples through a snail puppet. The puppet is able to represent emotions through physical movements made by the user.

The analyzed research works have the objective of detecting emotional states and use this information to make an explicit manifestation, of the emotions, by using software or electronic displays. However, the explicit interaction of users is needed in most of these works to indicate their emotional state. This characteristic can inhibit certain users to use the proposed approaches. In our proposal, the device automatically obtains the emotional state of the users by analyzing their comments on Facebook, and shows their emotions in real time without any user intervention. In this context, the detection of the emotional state is invisible for the user.

It is important to point out that our approach uses the one-dimensional valence concept to characterize emotions instead of multifaceted valence. One-dimensional valence implies characterizing an emotion as a pure positive or negative sentiment. In opposition to one-dimensional valence, multifaceted valence implies mixed feelings where feeling depends on the context [20]. Our selection is based on the current maturity of algorithms and methods to automatically detect emotions from text. The review of the literature about algorithms to predict positive, negative, and neutral polarities from non-structured texts (in a one-dimensional valence) indicates a precision about 60%–70%, which we consider a low precision level that needs to be improved. If these are the results for detection of one-dimensional valence, the prediction of mixed feeling could produce results too low to be significant.

## 3. The SWePT Service

As mentioned above, our proposed solution is composed of two contributions: (a) the web service called SWePT, which permits the detection of the polarity of texts; and (b) the Emotion-Bracelet, which shows the emotional state presented in the analyzed texts. In this context, the Emotion-Bracelet uses the SWePT web service which implements a hybrid approach for polarity detection of texts in Mexican Spanish. This approach combines the Sequential Minimal Optimization (SMO) algorithm with feature extraction from an affective lexicon. Therefore, the method requires a corpus and feature extraction from text. The corpus was developed using comments from Facebook and Twitter. An automatic extraction software system was implemented, and an affective lexicon in Mexican Spanish was created.

**Figure 1:**
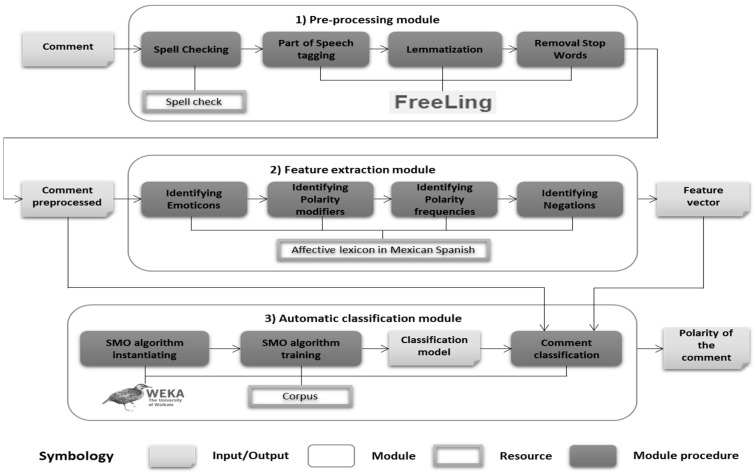
The modules of the SWePT method.

The SWePT service is divided into three main modules: pre-processing, feature extraction, and automatic classification. The two relevant resources created are: the affective lexicon in Mexican Spanish and the corpus. Figure 1 shows the execution flow of the modules and the resources generated.

(1) The pre-processing module: In this module each SNS comment is preprocessed to generate a comment that is free of spelling errors and stop words, and that is lemmatized and grammatically labeled. The FreeLing tool has been used for tagging, lemmatization, and removal of stop words.
(2) The feature extraction module: The goal of this module is to extract text features that provide information that is relevant to the detection of the polarity and intensity. The module takes into account such factors as denials, polarity modifiers, and polarity expressions. As a result, the process generates a feature vector that is based on the affective lexicon in Mexican Spanish.
(3) The automatic classification module: The goal of this module is to identify the polarity of the comments. The input of this module is a pre-processed comment and a feature vector that contains the number of positive, negative, and neutral words. This module was implemented using the Weka library [21] and the SMO algorithm. The output of this module is the polarity of the comment. The resources generated as part of the SWePT service are: an affective lexicon in Mexican Spanish and a corpus.

An affective lexicon in Mexican Spanish was created to obtain features that provide more information to the SMO algorithm. The steps followed for the creation of the affective lexicon are listed below:

Step 1. Manual translation from English to Spanish of words obtained from lexical resources. A set of words was translated (from English to Spanish) and labeled with their polarity and emotional category. This set of words was obtained from psychological theories [22,23,24,25,26,27,28].
Step 2. Manual enrichment based on semantic relationships. The set of words was enriched with semantic relationships: lexical families, inclusion relationships, and synonyms. The agreement among the annotators who manually labeled the corpus was evaluated with Krippendorff’s alpha. The subjectivity involved in the interpretation and annotation of the comments had an impact on the results.
Step 3. Manual enrichment of Mexican slang. The affective lexicon was enriched with Mexican slang and other expressions such as emoticons and interjections that are commonly used on Facebook and Twitter. The meaning and the semantic orientation of the expression were also added based on the context in which the word is used.

As a result, our lexicon has seven categories: negative emoticons (63); positive emoticons (68); polarity modifiers (97); interjections (57); and negations (15); words and phrases obtained from Facebook (255) and words labeled with polarity (3550). 

The corpus must be labeled with categories, and these categories should be defined based on the purpose of the corpus [29,30,31]. We classified our corpus into three categories to identify polarity (positive, negative, and neutral). Two categories were added to identify intensity (very positive and very negative).

## 4. The SWePT Service Implementation

**Figure 2:**
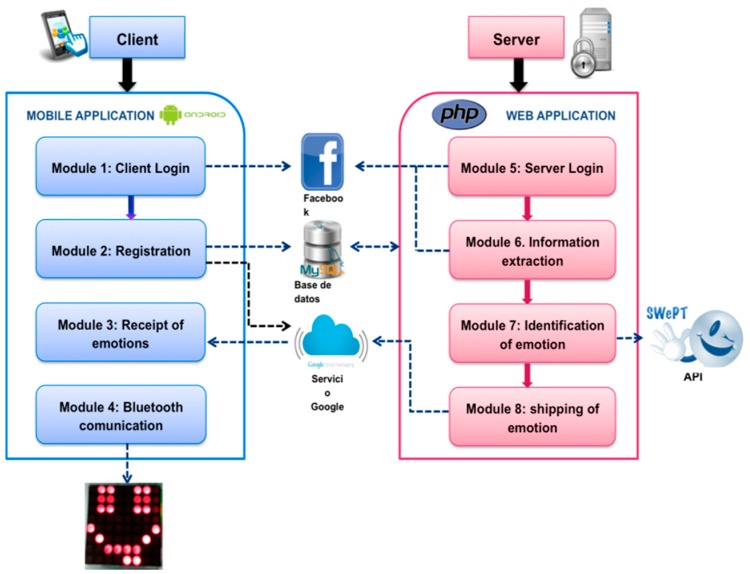
Client-Server Architecture of Emotion-Bracelet.

The implementation of the proposed approach allows the Emotion-Bracelet to express the polarity of the messages of a user that are posted on a SNS. Figure 2 presents the architecture of the Emotion-Bracelet. 

The developed system is a client-server application that is composed of hardware and software. 

For the hardware, the Emotion-Bracelet was built in order to express for the polarity using a LED matrix, which draws personalized emoticons for each emotion. For the software, the system was developed as a server of emotions, which works as a central node to identify the polarity sending information from a mobile device (as client). The polarity is shown to the user by a graphical interface. The software also directly interacts with the Emotion-Bracelet by communication via Bluetooth.

### 4.1. Client Application

**Figure 3:**
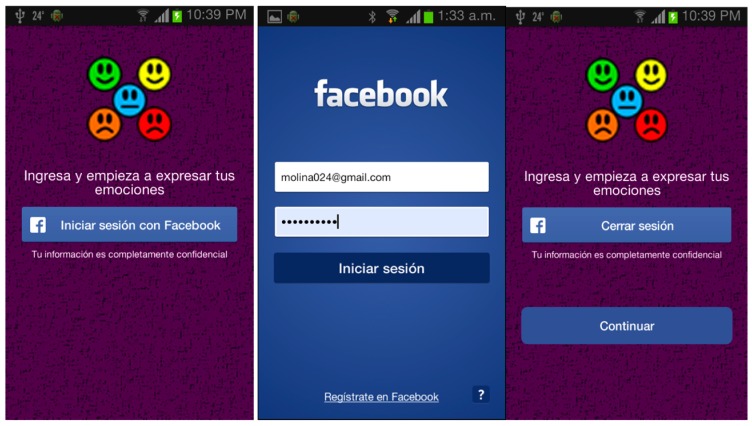
Application client in the mobile service client-server.

The client application is a mobile application that was developed for the android operating system. It runs from version 3.0 honeycomb (API 11) to the version 4.3 jelly bean MR2 (API 18). The software system was developed using the android developer tools (ADT), which are composed of the eclipse IDE, the ADT plugin, the Android SDK tools, the android platform-tools, the android platform, and the android Emulator. The modules of our client application are: 

Module 1. Client Login: This module carries out the connection with the Facebook social networking site in order to extract the information that identifies the user on Facebook. In order to enable the system to use the user information, this module also obtains the permissions for extracting posts written by the users. The graphical interface of this module is shown in Figure 3.

Module 2. Registration: This module carries out the recording of the user’s personal data in the database hosted on the server. Additionally, registration is carried out on the server of the service GCM in order to identify the mobile device when it sends the information about the identified emotion.

**Figure 4:**
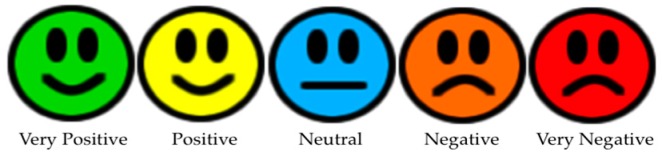
Emoticons.

Module 3. Receiving emotions: This module is a process that runs in the background on the mobile application whose goal is to receive and show the identified emotion to the user. When the mobile device receives an emotion, it is visualized using emoticon colors. Figure 4 shows these emoticons displayed on the mobile device.

Module 4. Bluetooth communication: This module enables the communication with the Emotion-Bracelet via Bluetooth. When the software application receives an emotion, the search process of the Emotion-Bracelet is started; if the Emotion-Bracelet is found, then the Bluetooth connection is established, and the value of emotion is sent to the bracelet. If the device is not found, then a vibration and an alert are sent to the user.

### 4.2. Server Application

The server application is a web application that was developed with the PHP programming language, which runs on the server every second. This application has the following features:

Module 5. Server Login: This module carries out the connection with the server application of Facebook SNS. 

Module 6. Information Extraction: This module allows us to extract user information through Facebook, which includes posts and comments. The Facebook query language (FQL) is used to extract the information from the Facebook database. This module runs automatically every second in order to monitor new texts in the profile of each user.

Module 7. Identification of Emotion: This module allows us to identify an emotion of the user by using the SWePT service. The input of the service is a text that represents the user’s comment, and the output is an array with a numeric value for the identified emotion: very positive, positive, neutral, negative, or very negative (see Table 1). 

Module 8. Sending of Emotions: The aim of this module is to send the value of the emotion identified to the mobile device. We use the messenger service of Google cloud (GCM) to reduce the consumption of the battery, memory, and Internet service of the mobile phone. This module queries the database to obtain the user ID and the emotion value. These values are sent through the service request to the GCM. When the GCM service receives this request, the mobile device sends information issued by the server.

## 5. The Design and Construction of the Emotion-Bracelet

The Emotion-Bracelet is a device that helps to create an emotional atmosphere among users who have difficulty expressing their emotions. The bracelet was designed with a weight and size that are discrete in appearance and that do not affect the daily activities of the user.

It is important to point out that one of the key aspects in developing the proposed approach was the definition of an electronic device to shown the emotions. One of the options we have analyzed is the use of smartwatches. This option has the advantage of having high availability devices with a technology extensively tested, and also with stable software applications. However, one of the disadvantages of this option is that most smartwatches have proprietary software and operating systems that make it difficult to install applications that use the smartwatch resources and data. Another drawback of this option is that most of these devices are too expensive. The option we follow was to develop our own electronic device with a specific functionality: to show the emotions of the user. This option has the advantage of having totally open software and operating systems that makes it easy having access to the data of the electronic display. Another of the advantages was the bracelet’s cost, which is about 30 dollars, compared with 150 dollars or more for a smartwatch.

**Figure 5:**
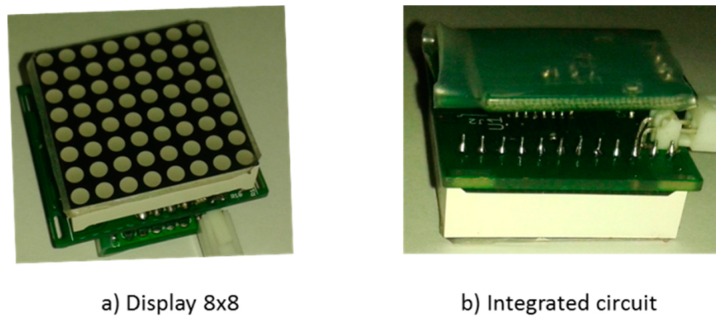
Design of the Emotion-Bracelet.

As a result, an 8 × 8 LED matrix was used for expressing the emotions, where every emotion is represented by a custom emoticon. The LED matrix allows a better perception of emotions between users. In addition to the LED array, a Bluetooth module HC-06 is used to perform communication with the mobile device, and a PIC16F628A microcontroller is used to communicate with the Bluetooth. The implementation of the bracelet is shown in Figure 5.

The flow of the Emotion-Bracelet and the mobile device starts when the mobile application receives the value of an emotion from the server. The mobile device automatically starts searching for the Emotion-Bracelet through Bluetooth and then establishes the communication; later it sends the value of the emotion to the bracelet. The PIC of Bracelet receives a byte of information from the Bluetooth and displays it in the matrix of LEDs as an emoticon. 

**Figure 6:**
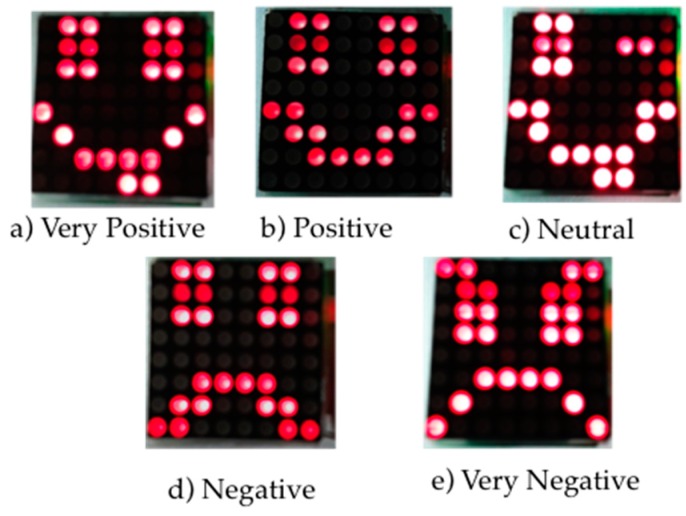
The emotions shown in the Emotion-Bracelet.

The emoticons that are represented in the LED matrix are divided into five categories of polarity of emotion: very positive, positive, neutral, negative, and very negative (see Figure 6).

### Case Study

To understand how the bracelet shows the moods of users and how it influences them, we examined several scenarios. Five subjects participated in the case study, which have signed an informed consent form to participate as volunteers in this research without economic compensation. The participants could leave the test at anytime without any penalties. We explained the use of the bracelet to the participants. We indicated that the bracelet was to be used for one day in public places and that the participants should post or send messages with their mobile device. 

At the end of the day, the participants filled out a questionnaire to report the impressions of the bracelet by people who saw the bracelet in action. Scenarios were carried out in public places when meeting with friends or strangers. It is important to point out that, in the case studies, we did not include people who had trouble identifying emotions, such as people with autism or mental disorders.

The following scenario shows the Emotion-Bracelet being used in a real situation; Jazmin is a 20-year-old female engineering student who is on an academic stay at the National Center of Research and Technological Development (CENIDET), which is a public research center in Mexico. Although Jazmin is a fictitious name, the case study was conducted with the students of CENIDET.

She accepted to use the Emotion-Bracelet and to post her emotions state on her social networking site. The first scenario was carried out in a public place, the central square of Cuernavaca City. In this squares, people walk, rest or play with children. Jazmin put the Emotion-Bracelet on and posted the following message “I am sure that this will be good”. The Emotion-Bracelet determined that this message had a very positive polarity and it showed the emoticon in Figure 6a. Some of the questions made by random people in the square who saw the Emotion-Bracelet were: why does this face means? What does this means? Do you need something? Afterwards, she posted the message “I was tired” to a friend. Immediately, the Emotion-Bracelet determined that the polarity of this message was negative and showed a negative emoticon (Figure 6d). Some other questions that some people asked her were: Can I help you? Are you ok? What is it?

For the Second scenario, Jazmin went to meet some of her friends. The Emotion-Bracelet was negative (Figure 6d). Then, when her friends saw the Emotion-Bracelet, they quickly asked her: Do you need a hug? Do you need to talk? Her friends provided support in order to solve her problems. Finally she posted “I think that this is working”. The Emotion-Bracelet changed to a positive emoticon (Figure 6b). 

The goal of this test was to analyze how the Emotion-Bracelet worked, the responses of the people when they saw the Emotion-Bracelet, and the responses of the users of the Emotion-Bracelet. 

## 6. Evaluation and Results

Two complementary evaluations were performed to the SWePT method and the electronic bracelet. The evaluations of SWePT method were done using standard metrics to evaluate algorithms for opinion mining, such as precision, recall, and F-measure. The precision measure shows what percentage of positive predictions where correct, whereas recall measures what percentage of positive events were correctly predicted. F-Measure which can be interpreted as a weighted average for the precision and recall. In the other hand, the evaluations for the Emotion-Bracelet were done analyzing the understanding (by people) of the faces shown in the Emotion-Bracelet. Besides, we analyzed the functionality of the bracelet. 

The SWePT method was evaluated taking into account seven features in order to identify the combination of features that better predicts the polarity of the messages: (a) Without any kind of pre-processing; (b) Grouping emoticons in two groups: positive and negative; (c) Pre-processing (spell checking, part of speech tagging, lemmatization, stop words removal); (d) Polarity frequencies (number of words or expressions classified as: very positive, positive, very negative or negative); (e) Polarity modifiers; (f) Negations; and (g) Emotions. The evaluation was carried out by using the 10-fold cross validation technique and the SMO algorithm implemented with the *Weka* library: without any kind of preprocessing; with pre-processing (spell checking, part of speech tagging, lemmatization, stop words removal); grouping emoticons in two groups (positive and negative); considering polarity frequencies (number of words or expressions classified as very positive, positive, very negative, or negative); polarity modifiers; negations; emotions.

The evaluation used two corpora of words; the first corpus was composed of 1500 comments, and the second was composed of 3100 comments. Each corpus was analyzed using five and three categories, where five categories imply very positive, positive, neutral, negative, and very negative, and three categories imply positive, neutral, and negative. Table 2 shows the results with the F-Measure which can be interpreted as a weighted average for the precision and recall. For more detailed of these evaluations, see [32]. These results indicate F-measure of the 83.4% of successful prediction of the polarity of the SWePT with the corpus of 1500 comments and 77.2% in the case of the corpus of 3100 comments.

Secondly, the Emotion-Bracelet was validated taking into account the understanding of people about the faces shown in the bracelet. The data collection was done through a questionnaire applied to 183 people, including both 105 men and 78 women between 15, and 45+ years of age. The results obtained through the questionnaire are shown in the Table 3. 

We analyzed the correct identification of the faces grouping the information into three categories (positive, negative, and neutral), and five categories (very positive, positive, neutral, negative, and very negative). The percentage of correct identification of the images was improved when the images were grouped into three categories, positive, negative, and neutral. The percentage of correct answers was similar between men and women. Also, the results revealed that the image used as neutral caused more confusion, as it was very low percentage of correct answers (19% and 38%, respectively). See Table 4.

We have compared the results of the questionnaires based on three categories (Positive, Neutral, and Negative) taking into account age and gender (see Table 5). 

**Figure 7:**
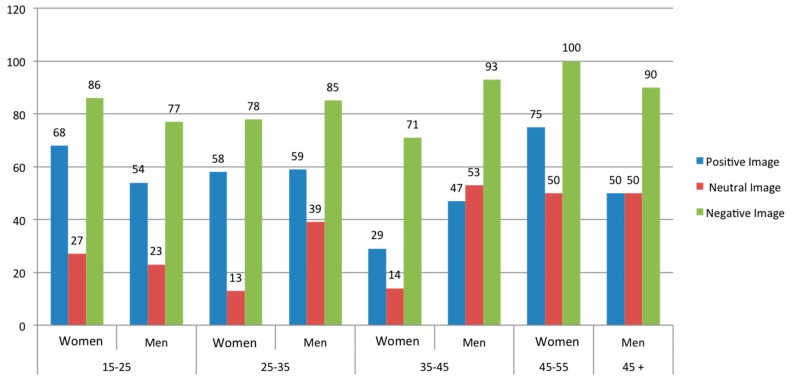
Comparative between women and men on the identification of images by their age.

In Figure 7, we can observe that women in the 15–25 age range identified all images better than men. The men in the 25–35 age range have identified all the images better than women. The women older than 45 years old identified the images better than men. Finally, both men and women older than 45 identified the images better than younger men and younger women.

Thirdly, the functionality of Emotion-Bracelet has been validated on several mobile devices using android versions from 3.0 to 4.3. The evaluation was conducted over a 50-day period with 15 Masters students from CENIDET. 

The students fulfilled the following requirements: (1) have an account on Facebook SNS; and (2) have a mobile device with android on version of 3.0 or higher; (3) sign an informed consent form to participate in this research. They could leave the test at anytime without any penalties.

Each student used the device for four hours on a workday, and they reported the results in a document where they validated the performance of the Emotion-Bracelet stating their experience with this new interface.

The procedure for validating the Emotion-Bracelet was the following; the user linked the mobile device to the Emotion-Bracelet and performed the required configuration actions. The number of posts on Facebook was free for each user. Each time that a user wrote a comment on Facebook, the extraction and identification modules were automatically executed. Later on, the identified emotion was notified by a push notification on the mobile device. When the emotion was received on the phone, the Bluetooth connection was automatically carried out with the Emotion-Bracelet in order to shown the corresponding emotion. Furthermore, the user could evaluate several characteristics of the Emotion-Bracelet: (1) the reception of a push notification each time that a comment was posted in Facebook; (2) the similarity between the emotion shown in the Emotion-Bracelet and the emotion shown in the mobile application; and (3) the Bluetooth communication between the mobile device and the Emotion-Bracelet interface. The authors validated that the Facebook comments were correctly extracted, identified, and stored in the database.

The tests showed a total of 211 Facebook comments. Of this total, 199 comments (96%) were considered in the evaluation, and 12 comments (4%) were considered to be failures. 

Overall, the use of the bracelet produced the following results. The participants found it fun to use a bracelet that directly showed their own moods. The most active users were young people; they were surprised by the emoticons showing the polarity of the messages. Generally, younger people do not have problems to show your mood using the bracelet.

In this study, we were able to confirm some of the conclusions of [33,34] that the closer the relationship between people, the easier it is to identify their moods. Nevertheless, another observation that we made was that even though face-to-face interaction could be more expressive in people who are in intimate [35] relationships, the bracelet helped people to recognize other people and their own moods.

## 7. Discussion and Conclusions

In this paper, the Emotion-Bracelet is presented as a new interface that allows users to express their emotions in real time by using non-intrusive mechanisms that are based on Facebook comments. The system automatically obtains user messages from Facebook, analyzes the text of the comment, and obtains the emotions expressed in the comment. With this information, the system sends the emotion to a LED Matrix via a Bluetooth connection and a specific emoticon is drawn on the device. This new interface can be applied in different contexts and for emotional communication, entertainment, health, family care, or education. 

The Emotion-Bracelet was also designed and developed using e-textile technology. The Emotion-Bracelet expressed emotions through emoticons represented in LEDs and distinguishes the change of emotion through the intensity of the colors represented.

We have developed a low-cost bracelet that allows showing the emotional states of a user. However, we have desisted to use a commercial bracelet because the high cost of these, as well as the dependence of the application of commercial devices.

We perform an evaluation of the SWePT system to measure the precision of the method to obtain the polarity. The evaluation was performed using standard metrics that compare the results of the polarity determined by the system with manual labeling of users that give a specific polarity for a large number of Facebook comments. Therefore, this evaluation permits determining the probability of the method to positive determine if the automatic evaluation of the method corresponds with the manual labeling. The results indicate that the better F-measure was obtained with the corpus of 1500 comments and evaluating three categories (positive, negative, and neutral, in this case we obtain an F-measure of 83.4%.

We also performed an empirical evaluation of the proposed approach to determine whether the proposed facial expression shown in the electronic display correspond with the feeling of a human user. To do this, we performed an extensive poll where people indicate an emotion for each one of the expressions shown in the electronic display. The results indicate that positive and negative images were correctly identified in more than 80%, however, the neutral image caused more confusion, and this was reflected with a low percentage of identification (19% and 38%).

The results indicate that, initially, the interface causes restlessness in the users. However, after several days, the Emotion-Bracelet starts to be assimilated and the users report that the use of the Emotion-Bracelet encourages the expression of emotions in an easy and funny way.

As future work, we plan to perform several tests to evaluate the impact of the system on the socialization of users.

## Figures and Tables

**Table 1:** Values of emotion.

Emotion	Value
Very Positive	1
Positive	0.5
Neutral	0
Negative	−0.5
Very Negative	−1

**Table 2:** Results with more efficient features combination.

Combination of Features That Give the Best Results	Corpus 1500 Comments		Corpus 3100 Comments	
	5 Cat.	3 Cat.	5 Cat.	3 Cat.
Features: (b) (c) (d) (e)	62.4%	83.4%	56.6%	77.2%

**Table 3:** Data obtained from questionnaires.

PARTICIPANTS	AGE	Num of Part	IMAGE 1 (VERY POSITIVE)								IMAGE 2 (POSITIVE)								IMAGE 3 (NEUTRAL)										IMAGE 4 (NEGATIVE)						IMAGE 5 (VERY NEGATIVE)					
			VP	%	P	%	N	%	Ng	%	VP	%	P	%	N	%	Ng	%	VP	%	P	%	N	%	Ng	%	VNg	%	N	%	Ng	%	VNg	%	N	%	Ng	%	VNg	%
WOMEN	15–25	22	10	45	10	45	2	9	0	0	3	14	15	68	4	18	0	0	12	55	4	18	6	27	0	0	0	0	1	5	19	86	2	9	0	0	6	27	16	73
MEN	15–25	26	7	27	15	58	4	15	0	0	5	19	14	54	7	27	0	0	15	58	5	19	6	23	0	0	0	0	4	15	20	77	2	8	0	0	5	19	21	81
WOMEN	25–35	45	17	38	22	49	6	13	0	0	14	31	26	58	5	11	0	0	21	47	15	33	6	13	2	4	0	0	7	16	35	78	3	7	2	4	8	18	35	78
MEN	25–35	54	22	41	24	44	6	11	2	4	14	16	32	59	5	9	3	6	19	35	13	24	21	39	1	2	0	0	5	9	46	85	3	6	2	4	9	17	43	80
WOMEN	35–45	7	2	29	4	57	1	14	0	0	3	43	2	29	1	14	1	14	3	43	3	43	1	14	0	0	0	0	2	29	5	71	0	0	1	14	0	0	6	86
MEN	35–45	15	6	40	4	27	5	33	0	0	4	27	7	47	3	20	1	7	2	13	4	27	8	53	1	7	0	0	0	0	14	93	1	7	1	7	5	33	9	60
WOMEN	45	4	1	25	2	50	1	25	0	0	0	0	3	75	1	25	0	0	1	25	1	25	2	50	0	0	0	0	0	0	4	100	0	0	0	0	2	50	2	50
MEN	45	10	2	20	6	60	1	10	1	10	3	30	5	50	2	20	0	0	2	20	2	20	5	50	0	0	1	10	0	0	9	90	1	10	0	0	1	10	9	90

Legend: VP: Very Positive; P: Positive; N: Neutral; Ng: Negative; VNg: Very Negative.

**Table 4:** Results of the analysis of questionnaires data.

Gender	3 Categories			5 Categories				
	P	N	Ng	VP	P	N	Ng	VNg
WOMEN	86%	19%	92%	38%	59%	19%	81%	76%
MEN	81%	38%	94%	35%	55%	38%	85%	78%

Legend: P: Positive; VP: Very Positive; N: Neutral; Ng: Negative; VNg: Very Negative.

**Table 5:** Comparative results of age between woman and men on the identification of images.

Gender	Age	P	N	Ng
Women	15–25	68%	27%	86%
Men	15–25	54%	23%	77%
Women	25–35	58%	13%	78%
Men	25–35	59%	39%	85%
Women	35–45	29%	14%	71%
Men	35–45	47%	53%	93%
Women	45+	75%	50%	100%
Men	45+	50%	50%	90%

Legend: P: Positive; N: Neutral; Ng: Negative.
